# *clusterMaker2:* a major update to *clusterMaker*, a multi-algorithm clustering app for Cytoscape

**DOI:** 10.1186/s12859-023-05225-z

**Published:** 2023-04-05

**Authors:** Maija Utriainen, John H. Morris

**Affiliations:** 1 Maastricht University, Maastricht, NL USA; 2 Department of Pharmaceutical Chemistry, University of California San Francisco, San Francisco, CA USA

**Keywords:** Clustering, Community detection, Network analysis, Visualization, Cytoscape

## Abstract

**Background:**

Since the initial publication of *clusterMaker*, the need for tools to analyze large biological datasets has only increased. New datasets are significantly larger than a decade ago, and new experimental techniques such as single-cell transcriptomics continue to drive the need for clustering or classification techniques to focus on portions of datasets of interest. While many libraries and packages exist that implement various algorithms, there remains the need for clustering packages that are easy to use, integrated with visualization of the results, and integrated with other commonly used tools for biological data analysis. *clusterMaker2* has added several new algorithms, including two entirely new categories of analyses: node ranking and dimensionality reduction. Furthermore, many of the new algorithms have been implemented using the Cytoscape *jobs* API, which provides a mechanism for executing remote jobs from within Cytoscape. Together, these advances facilitate meaningful analyses of modern biological datasets despite their ever-increasing size and complexity.

**Results:**

The use of *clusterMaker2* is exemplified by reanalyzing the yeast heat shock expression experiment that was included in our original paper; however, here we explored this dataset in significantly more detail. Combining this dataset with the yeast protein–protein interaction network from STRING, we were able to perform a variety of analyses and visualizations from within *clusterMaker2*, including Leiden clustering to break the entire network into smaller clusters, hierarchical clustering to look at the overall expression dataset, dimensionality reduction using UMAP to find correlations between our hierarchical visualization and the UMAP plot, fuzzy clustering, and cluster ranking. Using these techniques, we were able to explore the highest-ranking cluster and determine that it represents a strong contender for proteins working together in response to heat shock. We found a series of clusters that, when re-explored as fuzzy clusters, provide a better presentation of mitochondrial processes.

**Conclusions:**

*clusterMaker2* represents a significant advance over the previously published version, and most importantly, provides an easy-to-use tool to perform clustering and to visualize clusters within the Cytoscape network context. The new algorithms should be welcome to the large population of Cytoscape users, particularly the new dimensionality reduction and fuzzy clustering techniques.

## Background

High-throughput techniques to generate large proteomic, genomic, metabolomic and interactome datasets provide a wealth of information about basic biological processes as well as human diseases. More recently, with the advent of single-cell transcriptomics and spatially resolved proteomics, the size and complexity of these datasets have exploded. One of the techniques historically used for the analysis of high-throughput biological data has been clustering to categorize large numbers of data points into significantly smaller numbers of groups, where all of the members of the groups are similar or have similar features or behaviors. While many libraries and packages exist that implement various algorithms, there remains the need for clustering packages that are easy to use, integrated with visualization of the results, and integrated with other commonly used tools for biological data analysis.

Clustering algorithms have been used for over 2 decades to analyze microarray data [1], find complexes in protein–protein interaction data [2, 3], and more recently, to categorize single-cell data into cell types [4–6]. The goal of clustering algorithms is to group similar data together, but the definition of similarity may depend on the specific use case. For example, traditional hierarchical clustering of microarray data looks for similarity in expression patterns, while clustering of protein–protein interaction networks aims to group nodes based on how closely connected they are. Below, we discuss these two major approaches to clustering algorithms as well as dimensionality reduction and ranking approaches, with a focus on their application to nodes and edges in a network.

### Attribute clustering algorithms

Attribute clustering algorithms group nodes based on the similarity of the attributes of the nodes or a distance metric calculated using an edge weight. Examples of attribute clustering algorithms include hierarchical [1], k-means [7, 8], HOPACH [9], PAM [10], AutoSome [11], and Transitivity Clustering [12]. The results of these algorithms are generally presented as heatmaps, or dendrograms associated with heat maps (e.g., for hierarchical clusterings). The categories may be used to group nodes in a network context, but often they are used primarily as visualization aids since the groupings may be independent of the network topology.

### Network clustering algorithms

Network clustering algorithms find densely connected regions in a network. Examples of network clustering algorithms include AutoSOME [11], Affinity Propagation [13], Connected Components, GLay [14], MCODE [15], MCL [16, 17], SCPS [18], Transitivity Clustering [12], Leiden [19], Infomap [20], Fast Greedy [21], Leading Eigenvector [22], Label Propagation [23] and Multilevel [24]. Generally, these algorithms are used to break the network up into smaller groups, so rather than using heatmaps to visualize the results, the typical visualization is a clustered network, with multiple disconnected components.

Clustering algorithms such as MCL and Leiden are examples of discrete clustering. That is, the algorithm will place a node either in one group or another based on the parameters and the specifics of the algorithm. However, sometimes nodes are not clearly in one group or another, and, for example, might be strongly associated with more than one cluster. This can lead to an overinterpretation of the clustering results. Fuzzy clustering algorithms allow nodes to be a member of more than one cluster, where membership is a proportional value. For example, the fuzzy C-Means (FCM) algorithm [25, 26], which is (roughly speaking) the fuzzy analog to the k-means clustering algorithm, iteratively calculates cluster centroids and assigns nodes to clusters, repeating until some convergence criterion is reached.

### Dimensionality reduction algorithms

Dimensionality reduction techniques are used to reduce the number of input variables, that is, the dimensionality, in a dataset. The higher the dimensionality, the more complicated the analysis and modeling of the dataset becomes. Reducing the number of input variables is important, as the performance of algorithms like clustering and ranking can degrade with high dimensionality. Dimensionality reduction is a common technique and has been used for many years. Algorithms such as principal component analysis (PCA) [27], principal coordinate analysis (PCoA) [28], and multidimensional scaling (MDS) [29–31] are common and have been in use for many years. More recently, new techniques such as t-SNE [32] and UMAP [33] have been gaining popularity, particularly in the context of very large datasets such as single-cell transcriptomics, where both have been used to great effect to visualize cell-type clusters. Other dimensionality reduction techniques include non-negative matrix factorization (NMF) [34, 35], Isomap [36], Linear Embedding [37], and Spectral [38]. In the context of networks, embedding techniques can also be used as a form of layout algorithm to move nodes in a network based on a reduction of a dataset to two dimensions.

### Cluster ranking algorithms

The goal of ranking algorithms is to rank the clusters in the network by importance, where importance is measured by a provided attribute value (e.g., expression fold change) [39]. The ranking algorithms in *clusterMaker2* are: Hyperlink-Induced Topic Search (HITS), Multiple Attribute Additive Method (MAA), Multiple Attribute Multiplication Method (MAM), PageRank (PR) and PageRank with Priors (PRWP). The highest-ranking cluster most likely represents a biologically relevant grouping, for example, a cancer biomarker cluster. The more important the cluster, the higher rank it has.

### Cytoscape

Cytoscape [40–42] is open-source software used to analyze and visualize biological data. The Cytoscape 3 series was significantly refactored to improve overall performance and modularity. Cytoscape 3.0 was first released in 2013 and is now in its ninth release (Cytoscape 3.9). Cytoscape provides an extensive App application programming interface (API) that allows programmers to extend the native capabilities of Cytoscape with new functionality. The Cytoscape app store [43] currently lists over 350 apps, which extend Cytoscape in a number of ways, from implementing specific algorithms for targeted use cases, to integrating with public data repositories, to adding new visualization capabilities.

In addition to an API for the development of Apps, Cytoscape provides a mechanism called CyREST [44] that allows R and Python-based workflows to directly interact with Cytoscape networks and data and with Cytoscape apps that have exposed commands through Cytoscape automation [45].

The Cytoscape app store [43] currently lists over 30 apps that perform some kind of clustering. However, the user interfaces of these individual apps are very different, and there is no interaction between them. Further, there are other apps that would like to include various clustering algorithms without having to reimplement them.

### clusterMaker2

Here we present *clusterMaker2*, a significant update to the Cytoscape app *clusterMaker* [46]. In addition to clustering algorithms provided in the previous version of the app, a variety of frequently used dimensionality reduction techniques and ranking algorithms have been implemented in *clusterMaker2*. Furthermore, many of the new algorithms have been implemented using the Cytoscape *jobs* API, which provides a mechanism for executing remote jobs from within Cytoscape. The algorithms that use this technology calculate the results on a remote server and then send the results to *clusterMaker2* client. *clusterMaker2* is also automatable [45] through Cytoscape commands or CyREST [44], which allows it to be used by other apps in the Cytoscape ecosystem. For example, the stringApp [47] and the AutoAnnotate [48] app both use *clusterMaker2* as a clustering provider through the automation mechanism.*clusterMaker2* has added several new algorithms, including two entirely new categories of analyses: node ranking and dimensionality reduction. In the next several sections, we list the previous algorithms and describe any new algorithms.

## Implementation

*clusterMaker2* is an application available for use in the Cytoscape environment. Cytoscape is an open-source network visualization software platform. *clusterMaker2* extends Cytoscape by providing the functionalities needed for clustering, dimensionality reduction, and ranking. *clusterMaker2* is written in Java. *clusterMaker2* also provides new capabilities to use remote servers to execute algorithms asynchronously. In the next sections, we discuss relevant implementation details of each new algorithm, beginning with some details that cross all of the algorithms.

### Matrix API

For the algorithms implemented in Java, we realized that most if not all of the modern algorithms require some sort of matrix manipulation. To facilitate this and avoid duplication within the code, we implemented an internal matrix API that supports different backend implementations. Currently, the API supports a simple implementation of matrices as two-dimensional Java arrays, a faster implementation that uses the oj! Algorithms [49], and an implementation that includes sparse matrices from parallel colt [50]. In either case, convenience routines support the creation and manipulation of matrices from nodes and their attributes as well as edges.

### Remote (asynchronous) execution

Some of the network clustering and dimensionality reduction techniques described below were implemented using org.cytoscape.jobs package and a RESTful API. The Cytoscape jobs API provides a mechanism for executing jobs from within Cytoscape. It provides the framework for Cytoscape apps to marshal data, submit a remote job, check on the status of the submitted job, fetch the results, and unmarshal the data [49].

The front-end sends the graph data to the server and gets the analyzed data back. The options menu of each algorithm allows specifying a timeout period other than the default of 20 s, after which the algorithm shifts to running asynchronously in the background.

Falcon (https://falconframework.org/) is a Python-based web API framework for building app backends and microservices. clusterMaker2 uses Falcon to handle REST calls through Web Server Gateway Interface (WSGI).

### Automation

*clusterMaker2* exposes a wide variety of commands [45] that may be used through CyREST [44], including the RCy3 and py4cytoscape wrappers. *clusterMaker2* exposes four namespaces: **cluster** (Table 1), for network and node attribute cluster algorithms; **clusterdimreduce** (Table 2), for dimensionality reduction algorithms; **clusterrank** (Table 3), for cluster ranking algorithms; and **clusterviz** (Table 4) for the visualizations *clusterMaker2* provides. The *clusterMaker2* website (http://www.rbvi.ucsf.edu/cytoscape/clusterMaker2/) provides more details about the commands and their arguments.

**Table 1:** clusterMaker2 cluster commands

Namespace	Command	Description	Type
cluster	ap	Affinity propagation	Network clusterer
	autosome_heatmap	AutoSOME attribute clustering	Attribute clusterer
	autosome_network	AutoSOME network clustering	Network clusterer
	bestneighbor	Best neighbor filter	Cluster FIlter
	connectedcomponents	Connected components	Network clusterer
	cuttingedge	Cutting edge filter	Cluster FIlter
	density	Density filter	Cluster FIlter
	fastgreedy	Fast greedy (remote)	Network clusterer
	fcml	Fuzzy C-means cluster	Fuzzy cluster
	featurevector	Create correlation network from node attributes	Network clusterer
	fuzzifier	Cluster fuzzifier	Fuzzy cluster
	getcluster	Get an attribute cluster result	Utility
	getnetworkcluster	Get a cluster network cluster result	Utility
	glay	Community cluster (GLay)	Network clusterer
	hair_cut	HairCut filter	Cluster FIlter
	hascluster	Test to see if this network has a cluster of the requested type	Utility
	hierarchical	Hierarchical cluster	Attribute clusterer
	hopach	HOPACH-PAM cluster	Attribute clusterer
	infomap	Infomap (remote)	Network clusterer
	kmeans	K-means cluster	Attribute clusterer
	kmedoid	K-medoid cluster	Attribute clusterer
	labelpropagation	Label propagation (remote)	Network clusterer
	leadingeigenvector	Leading eigenvector (remote)	Network clusterer
	leiden	Leiden clusterer (remote)	Network clusterer
	mcl	MCL cluster	Network clusterer
	mcode	MCODE cluster	Network clusterer
	multilevel	Multilevel cluster (remote)	Network clusterer
	PAM Cluster	Partition around medoids (PAM) cluster	Attribute clusterer
	scps	Spectral clustering of protein sequences	Network clusterer
	transclust	Transitivity clustering	Network clusterer

**Table 2:** clusterMaker2 clusterdimreduce commands

Namespace	Command	Description
clusterdimreduce	isomap	Isomap (remote)
	lle	Local linear embedding (remote)
	mds	MDS (remote)
	pca	Principal component analysis
	pcoa	Principal coordinate analysis
	spectral	Spectral (remote)
	tsne	t-distributed stochastic neighbor
	tsneremote	tSNE (remote)
	umap	UMAP (remote)

**Table 3:** clusterMaker2 clusterrank commands

Namespace	Command	Description
clusterrank	HITS	Create rank from the hyperlink induced topic search algorithm
	MAA	Create rank from multiple nodes and edges (additive sum)
	MAM	Create rank from multiple nodes and edges (multiply sum)
	PR	Create rank from the PageRank algorithm
	PRWP	Create rank from the PageRankWithPriors algorithm

**Table 4:** clusterMaker2 clusterviz commands

Namespace	Command	Description
clusterviz	attributeview	Create new network from attributes
	clusterview	Create new network from clusters
	createRankingPanel	Show results from ranking clusters
	createResultsPanel	Create results panel from clusters
	destroyRankingPanel	Hide results from ranking clusters
	destroyResultsPanel	Destroy all cluster results panels
	heatmapview	JTree HeatMapView (unclustered)
	knnview	JTree KnnView
	linkSelection	Link selection across networks
	treeview	JTree TreeView
	unlinkSelection	Unlink the selection across networks

### Attribute clustering algorithms

In the latest version of *clusterMaker2*, we have added two new attribute clustering methods, PAM [10] and HOPACH [9]. These algorithms are similar to the existing k-means algorithm and were added by detailed transcoding of the original R implementation of HOPACH and an implementation of PAM based on the published description of the algorithm. Both were tested against R implementations of the algorithms.

### Network clustering algorithms

The following algorithms have been added in the latest version of *clusterMaker2*, using the remote execution mechanism described above: Leiden [19], Infomap [20], Fast Greedy [21], Leading Eigenvector [22], Label Propagation [23] and Multilevel clusterer [24]. In each case, the server-side implementation takes advantage of the python-igraph package for the algorithm itself (see Table 5).

**Table 5:** clusterMaker2 Cluster Algorithms. Italics algorithms are new since publication of the last paper

Type	Algorithm	Description	Source	Details
Attribute Clusterers	AutoSOME	The AutoSOME cluster algorithm [11]	The distributed AutoSOME implementation	Ported directly to clusterMaker by AutoSOME author
	*Correlation*	*Creates a correlation network based on node attributes*		
	Hierarchical	Standard hierarchical clustering as implemented by Eisen [1]	Cluster 3.0 package from Michiel de Hoon of the University of Tokyo	Ported by clusterMaker authors from C to Java
	k-means	Standard k-means clustering as implemented by Eisen [1] with the addition of silhouette estimation of k	Cluster 3.0 package from Michiel de Hoon of the University of Tokyo	Ported by clusterMaker authors from C to Java. Silhouette implemented by clusterMaker authors
	k-medoid	Modification of k-means from above to use medoid rather than means		Implemented by clusterMaker authors. Silhouette implemented by clusterMaker authors
	*HOPACH*	*Implementation of HOPACH* [9]* using PAM for the centroids*		*Implemented by clusterMaker authors*
	*PAM*	*Partition Around Medoids* [10]		*Implemented by clusterMaker authors*
Network	Affinity Propagation	The message passing-based approach to clustering by Frey and Dueck [13]	Implemented from the algorithm description in the original reference	Implemented by clusterMaker authors
	AutoSOME	The AutoSOME cluster algorithm [11]	The distributed AutoSOME implementation	Ported directly to clusterMaker by AutoSOME author
	*Fast-Greedy (remote)*	*The fast greedy modularity optimization algorithm for finding community structure *[21]	*python-igraph 0.9.8*	*Implemented on the server using igraph_community_fastgreedy*
	*Cluster Fuzzifier*	*An algorithm that takes an existing cluster and “fuzzifies” it*		*Developed and implemented by clusterMaker authors*
	Community (GLay)	Newman-Girvan [51] community clustering as implemented by Su et al [14]	The original GLay plugin for Cytoscape	Ported by clusterMaker authors
	Connected Components	Simple division based on connectivity		Implemented by clusterMaker authors
	*Fuzzy C-Means cluster*	*An implementation of FCM* [25, 26]		*Implemented by clusterMaker authors*
	*Infomap (remote)*	*Finds the community structure of the network according to the Infomap method* [20, 52]	*python-igraph 0.9.8*	*Implemented on the server using igraph_community_infomap*
	*Leiden (remote)*	*Finds the community structure of the graph using the Leiden algorithm* [19]	*python-igraph 0.9.8*	*Implemented on the server using igraph_community_leiden*
	*Label Propagation (remote)*	*Finds the community structure of the graph according to the label propagation method* [23]	*python-igraph 0.9.8*	*Implemented on the server using igraph_community_label_propagation*
	*Leading Eigenvector (remote)*	*Newman's leading eigenvector method for detecting community structure* [22]	*python-igraph 0.9.8*	*Implemented on the server using igraph_community_leading_eigenvector*
	MCL	Markov clustering algorithm from van Dongen [16, 17] that uses random-walks to simulate flow	Implemented from original thesis with reference to C implementation for validation of results	Implemented by clusterMaker authors as a parallel algorithm to take advantage of multiple CPU cores
	MCODE	Bader and Hogue [15] algorithm for finding modules in PPI networks	The MCODE Cytoscape plugin	Ported by clusterMaker authors
	*Multilevel Cluster (remote)*	*Community structure based on the multilevel algorithm* [24]	*python-igraph 0.9.8*	*Implemented on the server using igraph_community_leading_multilevel*
	SCPS	Spectral clustering algorithm for BLAST similarity networks [18]	Implemented from the algorithm description in the original reference using the authors’ implementation to validate results	Implemented by clusterMaker authors
	Transitivity Clustering	Transitivity based clustering approach from Wittkop et al. [53]	Ported from Cytoscape TransClust plugin	Ported by original TransClust authors
Ranking	*MAA*	*Multiple nodes and edges (additive sum)*	*Implemented as part of Ranklust* [39]	*Added by Ranklust author*
	*MAM*	*Multiple nodes and edges (multiply sum)*	*Implemented as part of Ranklust* [39]	*Added by Ranklust author*
	*PRWP*	*PageRankWithPriors algorithm*	*Implemented as part of Ranklust* [39]	*Added by Ranklust author*
	*PR*	*PageRank algorithm*	*Implemented as part of Ranklust* [39]	*Added by Ranklust author*
	*HITS*	*Hyperlinked InducedTopicSearch algorithm with priors*	*Implemented as part of Ranklust* [39]	*Added by Ranklust author*
Dimensionality reduction	*Isomap (remote)*	*Implementation of the Isomap (Isometric Mapping) algorithm* [36]	*scikit-learn 0.24.2*	*Implemented on the server using sklearn.manifold.Isomap*
	*Locally Linear Embedding (remote)*	*Implementation of the Locally linear embedding (LLE) algorithm* [37]	*scikit-learn 0.24*	*Implemented on the server using sklearn.manifold.LocallyLinearEmbedding*
	*MDS (remote)*	*Implementation of classic multidimensional scaling* [29–31]	*scikit-learn 0.24*	*Implemented on the server using sklearn.manifold.MDS*
	*Principal Component Analysis*	*Implementation of Principal Component Analysis*		*Implemented by clusterMaker2 authors. using OjAlgo library version 49.2.1 to calculate the eigenvalues*
	*Principal Coordinate Analysis*	*Implementation of Principal Coordinate Analysis (MDS)*		*Implemented by clusterMaker2 authors. using OjAlgo library version 49.2.1 to perform SVD*
	*Spectral (remote)*	*Implementation of spectral embedding* [38]	*scikit-learn 0.24*	*Implemented on the server using sklearn.manifold.SpectralEmbedding*
	*t-Distributed Stochastic Neighbor*	*Local implementation of t-distributed Stochastic Neighbor Embedding* [32]* (t-SNE)*	*Leif Jonsson’s Java implementation of t-SNE*	*Ported to clusterMaker2 by clusterMaker authors*
	*tSNE (remote)*	*Implementation of t-distributed Stochastic Neighbor Embedding* [32]* (t-SNE)*	*scikit-learn 0.24*	*Implemented on the server using sklearn.manifold.TSNE*
	*UMAP (remote)*	*Implementation of Uniform Manifold Approximation and Projection (UMAP)* [33]	*umap-learn 0.5.1*	*Implemented on the server using umap.UMAP*

As discussed above, sometimes nodes are not clearly in one group or another, and, for example, might be strongly associated with more than one cluster. We introduced two fuzzy clustering algorithms, which allow nodes to be a member of more than one cluster and where membership is a proportional value. The first of these is the well-known Fuzzy C-Means (FCM) algorithm [25, 26], which we implemented in Java based on the published method. The second fuzzy algorithm, which we call a cluster “fuzzifier,” was developed and implemented by the *clusterMaker2* authors. It is based on the observation that the most expensive part of the fuzzy c-means calculation is the iterative determination of the centroid of each cluster. *clusterMaker2* already provides several algorithms to provide a first-round segregation of nodes into clusters, and given those clusters, it is relatively easy to calculate a centroid and then evaluate proportional measurement based on those centroids. The cluster “fuzzifier,” then, takes as its input a previous clustering performed by *clusterMaker2*, such as MCL, and then “fuzzifies” it by reassigning nodes to clusters proportionally. The algorithm assumes that edge values exist and are distances (not weights) and then calculates the distance from each node to each cluster centroid using a Java implementation of the algorithm defined by the R usedist [54] package’s distance_to_centroid function.

### Dimensionality reduction algorithms

Some of the dimensionality reduction techniques in *clusterMaker2* are coded in Java and implemented completely in the app, whereas others are implemented partially using the Cytoscape API and partially on the server. The Java dimensionality reduction techniques in *clusterMaker2* are: PCA, PCoA, and t-SNE [32]. The ones using REST implementations are: Isomap [36], Linear Embedding [37], MDS [29–31], Spectral [38], t-SNE [32], and UMAP [33]. t-SNE has been implemented in both ways.

### Cluster ranking algorithms

MAA and MAM are simple algorithms that through addition or multiplication calculate the average score of a cluster [47]. The remaining methods utilize network ranking algorithms from the Java JUNG library [48]. All of the algorithms except HITS use node and/or edge values to calculate the rank of each cluster. All of these algorithms have been implemented directly in Java.

### Visualization

The latest version of *clusterMaker2* adds two new visualizations and a new visual tool that augments others, in addition to the three visualization types previously described [46] (see Figure 1 in [46]).

**Fig. 1:**
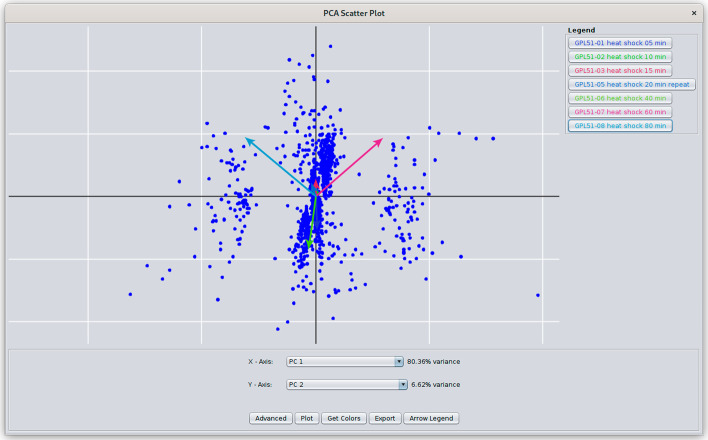
PCA scatter plot from *clusterMaker2.* This plot shows the two principal components of the yeast heat shock data set (GPL51) discussed in the paper. The arrows represent the loading vectors for each of the attributes. The legend on the right shows the attributes and their loading vector colors, which may be changed by clicking on the button. Selection is bi-directional, so selecting a point in the scatter plot will select the corresponding node in the network. See the Fig. 2 legend for a description of the **Plot**, **Advanced**, and **Get Colors** buttons

First, we added a traditional PCA plot, complete with loading vectors, as shown in Fig. 1. By adjusting the colors and transparency, users can see the variance and where the major contributions to that variance come from. The dataset in the figure is from an early microarray experiment on the stress response in yeast [55]. The plot allows users to pan and zoom, and to map colors from the Cytoscape network onto the points on the plot.

**Fig. 2:**
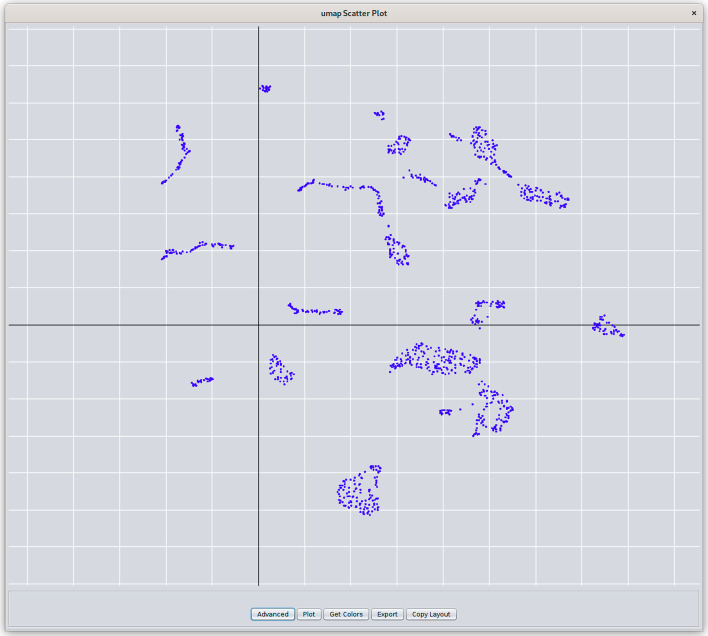
UMAP scatter plot from *clusterMaker2*. Similar to the PCA plot shown in Fig. 1, UMAP, tSNE, and other dimensionality reduction techniques produce scatter plots with the same functionality except that there are no loading vectors. The plots may be pan and zoomed and selection is also bi-directional. Hovering over a node will show it’s name, and the **Get Colors** button can be used to color the points according to the color of the corresponding node in the network. The **Advanced** button allows the user to select the point size and color. The use can **Export** the plot as a PNG, JPG, SVG, or PDF file. The **Copy Layout** button will use the X, Y coordinates of the points in the scatter plot to move the corresponding nodes in the network

For other dimensionality reduction techniques and embeddings, a similar scatter plot but without loading vectors is used. For example, in Fig. 2, the same data as in Fig. 1 is embedded using UMAP and shown in a scatter plot. The non-PCA scatter plots provide an additional feature where the 2D coordinates of the points on the plot may be mapped onto the Cytoscape network. This provides a dimensionality reduction-driven layout.

Finally, we added the ability to browse network clustering results in the Cytoscape Results Panel. This includes a small thumbnail of each cluster and allows the user to select all of the nodes belonging to that cluster by clicking on the row. The Results Panel also provides some summary information and a column for an algorithm-specific measure of cluster quality. In addition, the overall modularity of the clusters is reported as part of the side panel.

### Implementation details for all algorithms

Table 5 shows the algorithms, their descriptions and other details, and sources.

## Results

As described above, *clusterMaker2* provides a variety of clustering, dimensionality reduction, and ranking algorithms to explore biological networks and attributes. In this scenario, we will show how these various algorithms can be used together to explore a dataset, and in addition, we will demonstrate the advantages associated with having *clusterMaker2* as part of the Cytoscape ecosystem and thus able to integrate with other Cytoscape apps and capabilities.

Our scenario begins with data from a yeast heat-shock experiment conducted by Gasch and colleagues, but not reported on in their paper [55]. We then combine this data with yeast protein–protein interaction data from the STRING database. The end result is a network of yeast proteins and their physical interactions annotated with transcriptional changes resulting from heat shock.

To explore this data, we cluster the network to find complexes and other tightly connected proteins, then cluster the expression data to find expression patterns, and group genes together based on those expression patterns. Using the expression patterns to color the nodes in the network allows viewing the transcriptional patterns within the context of the protein complexes and groupings. Given the somewhat manual nature of the approach taken to group the expression patterns, we explore the use of dimensionality reduction techniques to see if we could have grouped the expression data more easily that way.

Next, we explore a subset of the most significant protein clusters using our cluster “fuzzifier” to determine if there might be associations between the clusters that are lost as a result of the discrete nature of the cluster algorithms. Finally, we rank the protein clusters using the transcription data to determine which of these clusters have the most significant response to heat shock at the transcriptional level.

### Data sources

Data from two sources are used in this scenario: the physical protein–protein interaction network for *Saccharomyces cerevisiae* from the STRING database, and the same heat shock data from Gasch et al. [55] that we used in our previous paper [46]. We extracted this dataset from GEO series GSE18 platform GPL51 and used the matrix data directly. In particular, we used the following columns: “GPL51-01 heat shock 05 min”, “GPL51-02 heat shock 10 min”, “GPL51-03 heat shock 15 min”, “GPL51-05 heat shock 20 min repeat”, “GPL51-06 heat shock 40 min”, “GPL51-07 heat shock 60 min”, and “GPL51-08 heat shock 80 min.”

### Importing the protein–protein interaction (PPI) network

**Fig. 3:**
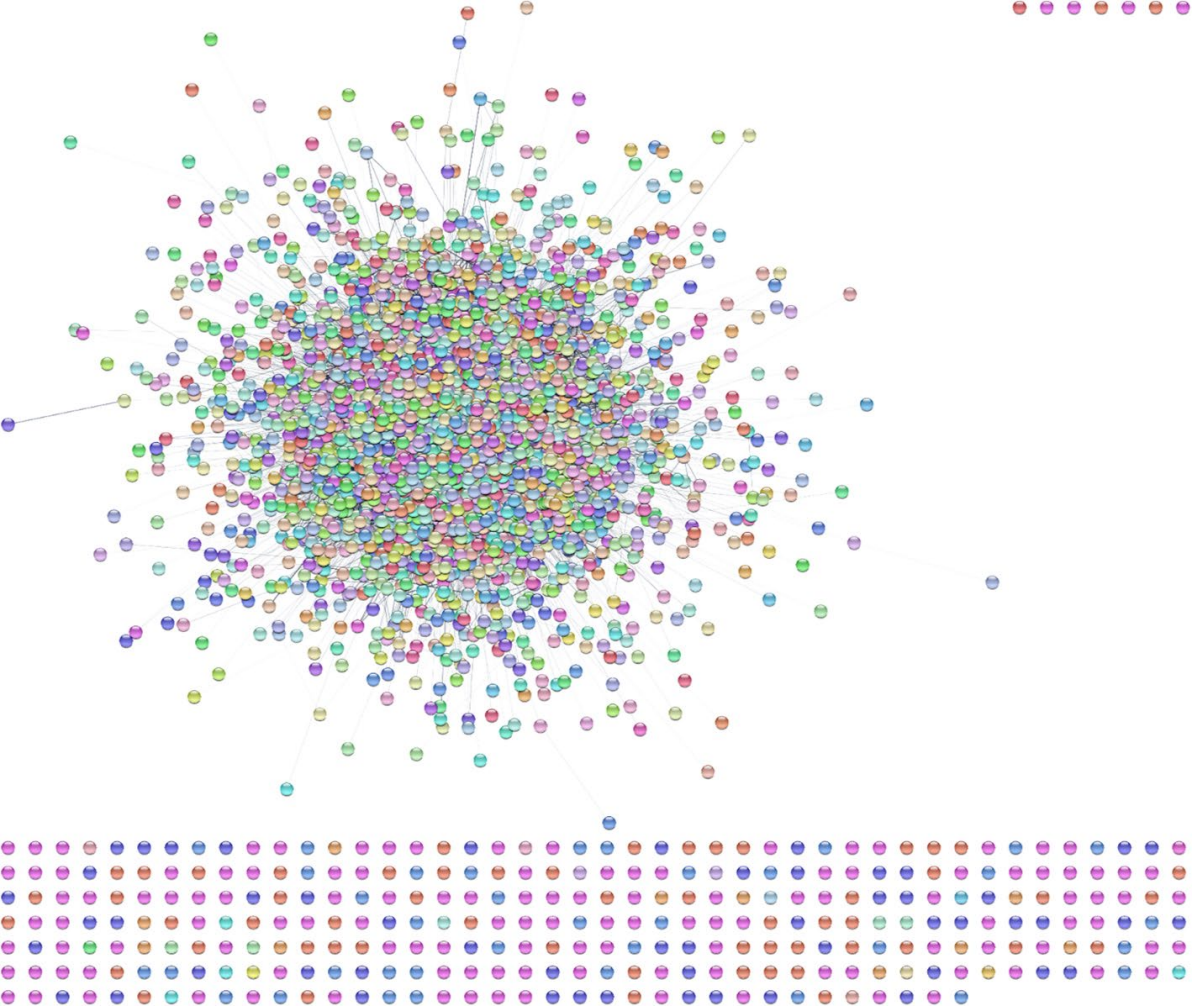
Cytoscape export of the STRING protein–protein interaction network for Saccharomyces cerevisiae imported using the stringApp. The confidence score used was 0.5 and the standard STRING style is shown

We begin by using the stringApp [47] to load the genome-wide *Saccharomyces cerevisiae* physical protein–protein interaction network into Cytoscape. This is accomplished by pulling down the **File** menu and selecting **Import → Network from Public Databases…** Select **STRING: protein query** as the Data Source and ***Saccharomyces cerevisiae*** as the species. Then select “All proteins of this species” and click on “physical subnetwork” under **Network type**. Set the **Confidence (score) cutoff:** to 0.50 to get a network of reasonable size without too many possible false-positive edges. Now, clicking on “Import” will import the entire yeast protein–protein interaction network (Fig. 3).

### Importing the expression data

To Import the expression data from GPL51 described above, we dropped any cells from the data matrix with |log_2_(fold change)|< 1, and any rows (genes) that did not have more than one significant fold change in any of the heat-shock data columns mentioned above.

Once the dataset was modified as above, we imported it into Cytoscape using **File → Import → Table from File.** To map the data in the expression file to the PPI network, we set the “Key column for the network” to **display name** and under “Advanced Options…” we set the delimiter to **Tab** and turn off **COMMA**. Now we have a PPI network where each of the proteins are annotated with the 5-min (GPL51-01), 10-min (GPL51-02), 15-min (GPL51-03), 20-min (repeat) (GPL51-05), 40-min (GPL51-06), 60-min (GPL51-07), and 80-min (GPL51-08) heat-shock expression fold changes, if they passed our cutoffs. We are now set to analyze this dataset.

### Clustering the PPI network

The PPI network as shown in Fig. 3 is too dense for easy interpretation. A typical next step is to break the network up into clusters, which represent tightly connected groups of proteins such as complexes. We will use Leiden clustering [19] to do this. The Leiden algorithm is an improvement of the Louvain algorithm, which maximizes the modularity score of each community by comparing how much more connected the nodes are in a community compared to a random network. It has three phases: local moving of nodes, refinement of the partition aggregation of the network based on the refined partition, and using the non-refined partition to create an initial partition for the aggregate network.

**Fig. 4:**
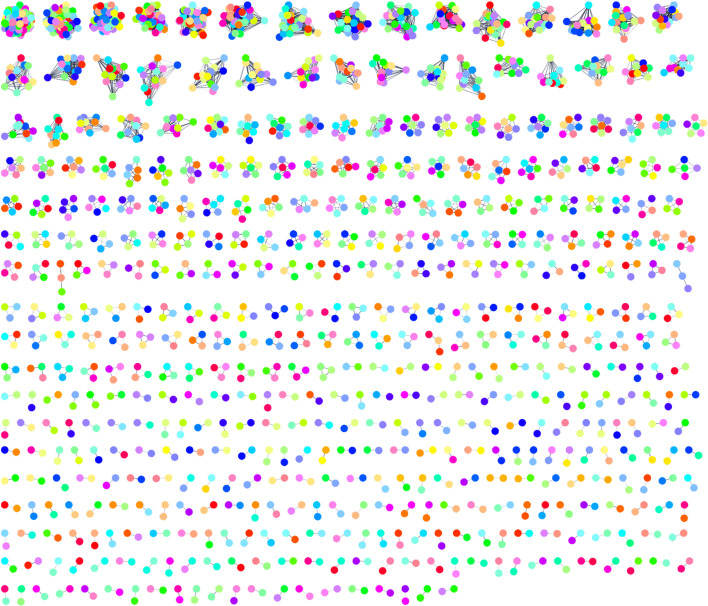
Export of the *Saccharomyces cerevisieae* protein–protein interaction network after clustering with Leiden clusterer using a resolution of 0.5 and 30 iterations. Node colors are random (preserved from the initial STRING network), and we have disabled the “glass ball” effect as well as the STRING-style labels using the stringApp results panel

Select **Apps → clusterMaker Cluster Network → Leiden Clusterer (remote)** to bring up the Leiden cluster options. After some experimentation (Leiden clustering is relatively quick), we found a resolution parameter of 0.5 and 30 iterations to work well. The resolution parameter correlates with the size of the communities. Higher resolution parameter values lead to smaller communities, while lower values lead to fewer, larger communities. The number of iterations gives the number of times Leiden algorithm is iterated. Each iteration improves the partition further. Adjusting the parameters to be the ones given above results in a network clustered to a biologically meaningful extent. For the **Source for array data**, select “stringdb::score” as the Attribute. This is the edge confidence score assigned by STRING. Select “Create new clustered network” and click “OK”. The resulting network should look similar to Fig. 4. Note that we have disabled the “Glass ball effect” and “STRING style labels” in the STRING results panel at the right. A quick exploration of the clustered network confirms that Leiden has done a reasonable job—the first four clusters in the upper left-hand corner are the ribosome, preribosome, large subunit of the preribosome, and the mitochondrial ribosome, respectively. This makes sense, as these are all large complexes. The fifth cluster is RNA polymerase II holoenzyme, and the sixth is the spliceosome. What complex a cluster represents can be determined by selecting a cluster and running the stringApp functional enrichment on that cluster using the entire genome as a background.

### Hierarchical clustering of expression data

A classical analysis of an expression dataset would involve performing a hierarchical clustering of the data and viewing it using a heatmap with associated dendrogram. In hierarchical clustering, at each iteration of the proximity matrix the similar clusters merge with other clusters until one cluster is formed. The nodes the most similar to each other are grouped together earlier.

**Fig. 5:**
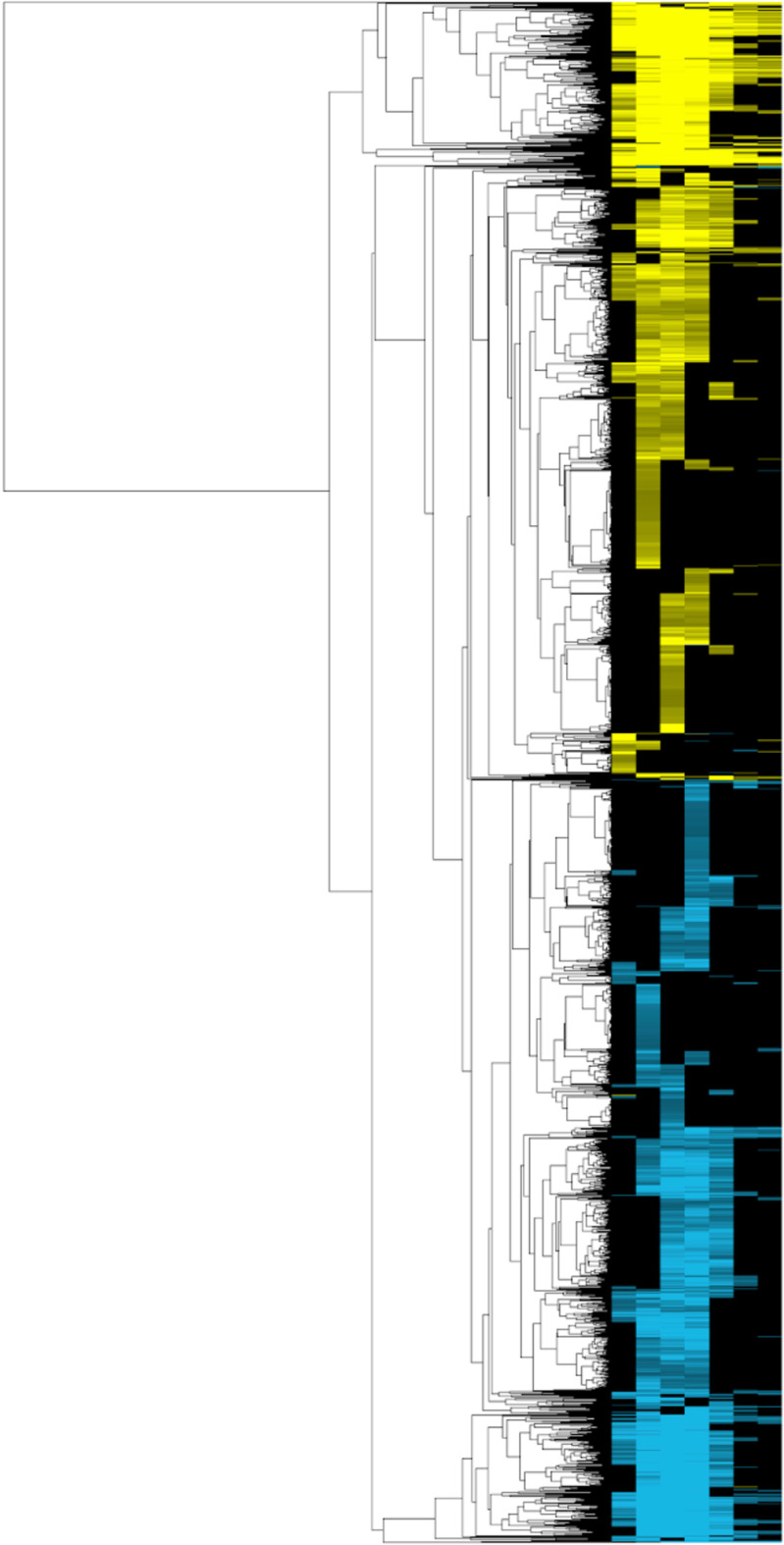
Export from the clusterMaker2 Jtree HeatMap view of the hierarchical clustering of heat shock data from GPL51. The image uses a standard yellow-cyan color scheme where yellow grandient indicates the degree of over-expression and cyan gradient indicates the degree of under-expression

To do this using *clusterMaker2*, we select **Apps → clusterMaker Cluster Attributes → Hierarchical cluster**. We then select all of the heat shock columns (GPL51-01 heat shock 5 min, GPL51-02 heat shock 10 min, GPL51-03 heat shock 15 min, GPL51-05 heat shock 20 min repeat, GPL51-06 heat shock 40 min, GPL51-07 heat shock 60 min, and GPL51-08 heat shock 80 min), then click on “Show TreeView when complete” and finally click OK. This will bring up a heat map with the associated dendrogram for the dataset (Fig. 5). By selecting branches of the dendrogram, we can select groups of genes in the heatmap and simultaneously select the corresponding proteins in the PPI view.

### Coloring the PPI network

To help understand the biological significance of these transcriptional changes at the protein level, we would like to find a mapping from the hierarchical clustering onto the proteins in the PPI network. This could be useful, for example, to see if any complexes are particularly affected by transcriptional changes.

**Fig. 6:**
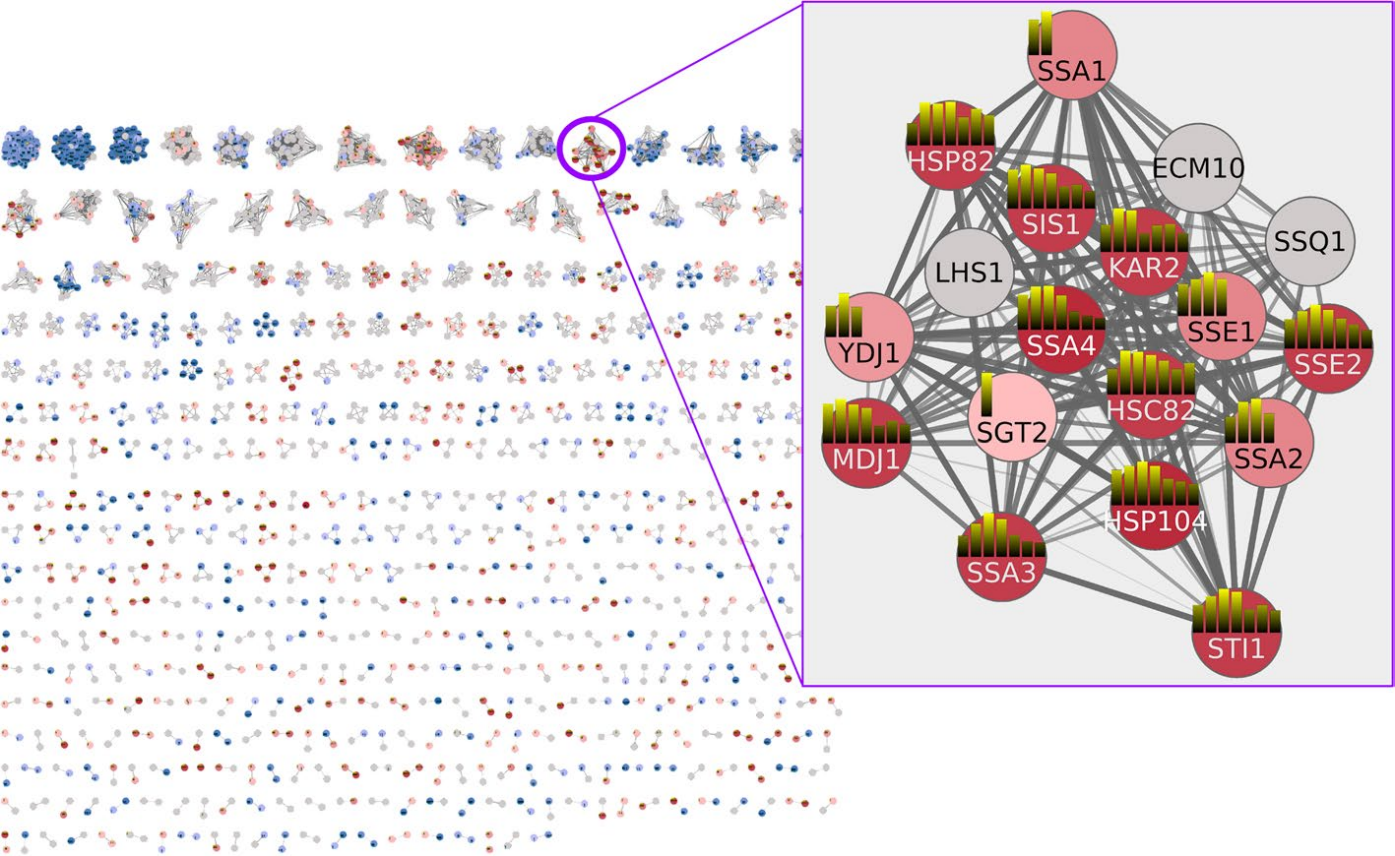
Using colors and heatstrips to explore PPI and expression data. Nodes are colored using a BrewerColor Red-Blue palette derived from the **Color** column as described in the text. Red indicates overexpression and blue indicates underexpression. The inset shows proteins involved in protein folding and refolding are overexpressed during heat stress response. The heatstrips (small bar charts on the nodes) show the expression changes at the individual time points and use the yellow-cyan gradient described in Fig. 5

There are two ways to map expression dataset onto the network. The simplest approach is to use the **Map Colors Onto Network** capability in TreeView: select all of the rows and click on “Create HeatStrips.” This will add bar charts showing the expression fold changes at the various times post-heat-shock on the nodes (as in the Fig. 6 inset). Unfortunately, this is extremely hard to see when looking at the entire network. To explore this dataset more fully, we used the ability to select branches of the dendrogram, which selected the corresponding nodes in our PPI. We created a new column in the Node Table named **Color** and assigned values from -10 to 10 depending on the level of upregulation or downregulation across time points we saw in the corresponding dendrogram branch. This was done quite crudely based only on visual inspection of the dendrogram branches and heatmap colors. For completeness, we did try to calculate a cut of the tree using external tools, but the dendrogram is extremely dense and it was difficult to achieve satisfactory results that provided anything close to a usable distribution on the tree.

To map the colors, we created a continuous mapping in Cytoscape using the ColorBrewer [56] diverging Red-Blue palette. The combination of the approximate color and heatstrips provides a general overview of the expression changes for each cluster, and when we zoom in, the corresponding details (Fig. 6). Note that SSA1, a member of the HSP70 family, is initially upregulated for the first 10 min, but shows no expression change after that. This can be seen easily in the close-up view (or the corresponding row in the heatmap), but is not visible in the PPI overview.

### UMAP analysis of expression data

The manual process we used above to choose colors was relatively straightforward, albeit somewhat time-consuming. As the number of attributes increases, however, it can be extremely difficult to group the genes together in meaningful ways. Dimensionality reduction techniques have become an increasingly valuable tool to group data based on numerous attributes by providing a visualization that groups similar items together even when the number of attributes is very large.

We will now look at the same heat shock expression data used for the hierarchical clustering Fig. 5, but this time using the Uniform Manifold Approximation and Projection [57] (UMAP) approach to explore a 2D embedding of this multidimensional data. UMAP can be used for visualization of high-dimensional datasets similarly to t-SNE, but also for general nonlinear dimension reduction. In mathematical terms, it is a manifold learning technique constructed from the theoretical framework based on Riemannian geometry and algebraic topology. It can use labels for supervised dimensionality reduction and transform new data into a pretrained embedding space.

**Fig. 7:**
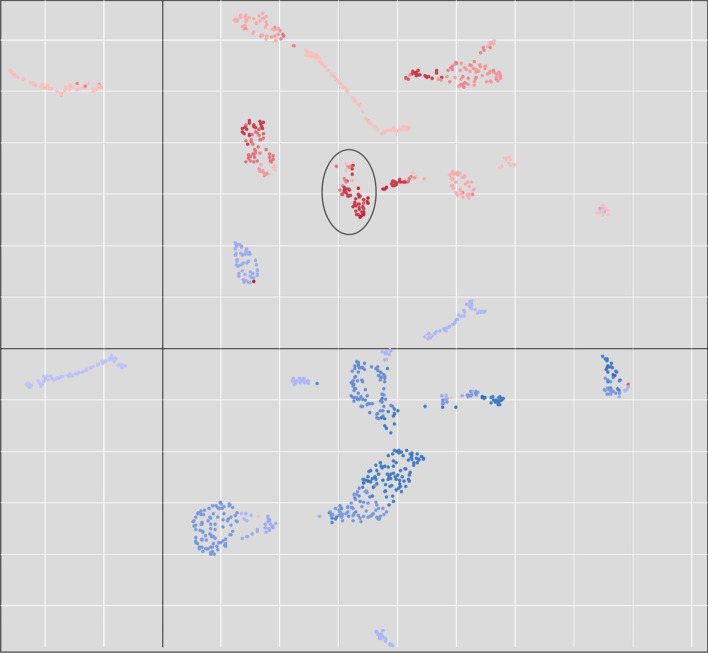
UMAP embedding of the heat shock expression data exported from clusterMaker2’s scatter plot (see Fig. 2). The colors correspond to the manually created **Color** column where fold change values are mapped onto the Red–Blue ColorBrewer gradient (red = degree of overexpression, blue = degree of underexpression). The circled group is enriched in protein folding, unfolded protein binding, and protein refolding

Because there are a number of nodes with no data, first select all of the nodes with a color value or a “Non-Zero Count” 1 or greater (you can sort the column or use the Cytoscape Filter tab). Once they are all selected, use **Apps → clusterMaker Dimensionality Reduction → UMAP (remote)** to bring up the UMAP options. Select all six of the heat shock columns, select “Only use data from selected nodes” and set the “Number of neighbors” to 20 and the “Minimum distance” to 0.5. The number of neighbors controls how UMAP balances local versus global structures. It constraints the size of the local neighborhood the algorithm looks at when learning the data. Low number of neighbors makes UMAP concentrate on a local structure and potentially lose the big picture, whereas larger values of number of neighbors results in UMAP looking at larger neighborhoods with the potential cost of losing fine details. The minimum distance parameter controls how tightly UMAP packs points together. It provides the minimum distance that points are allowed to be apart. Low values of minimum distance will result in clumpier embeddings, which serve the interest in finer details better. Larger values prevent UMAP from packing points together and puts the focus on the preservation of the topological structure. Make sure to also click “Show scatter plot with results” so we can explore the resulting embedding. Click OK to send the request to the server to perform the UMAP. Once the UMAP scatter plot comes up, click on “Get Colors” to apply the red-blue coloring from the nodes to the UMAP. It is easy to see how well the UMAP segregates the data (Fig. 7). Highlighting the red group in the center and performing an enrichment on the selected nodes indicates significant enrichment for protein folding. Indeed, many of the nodes in this cluster are part of the highlighted group in Fig. 6.

### Fuzzy clustering

Fuzzy clustering can help to reveal the relationships between clusters to find instances where proteins are shared between clusters. As described above, we have implemented a novel approach to calculating fuzzy clusters in *clusterMaker2*, which we call a “fuzzifier.”

**Fig. 8:**
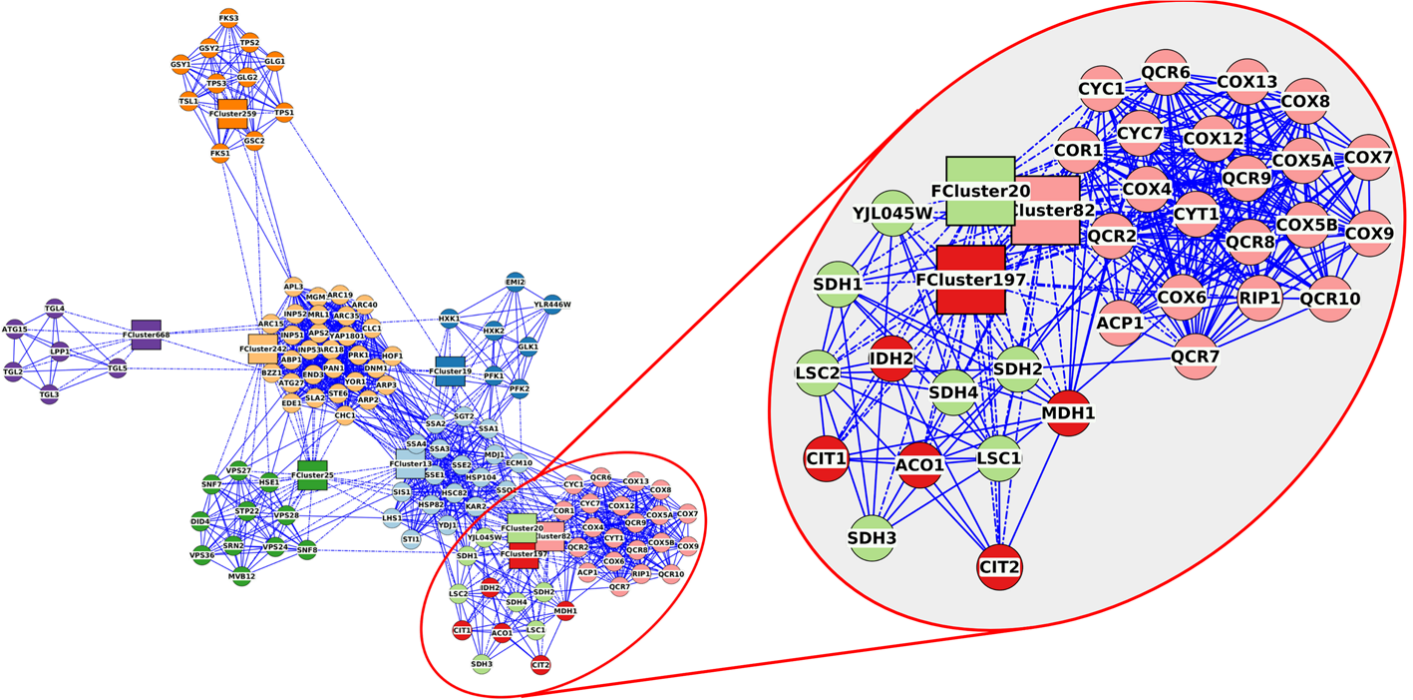
Fuzzy clustering of 9 selected Leiden clusters. Inset shows three intertwined clusters that represent mitochondrial proteins. Solid lines are from the original STRING protein–protein interaction network and dashed lines are the membership edges between proteins and their fuzzy-cluster centroid. Node colors are assigned based on cluster number, and each fuzzy cluster is represented by a FClusterNNN node where the NNN is replaced by the cluster number of the dominant cluster. The FCluster node is put at the centroid of all nodes in that fuzzy cluster

To apply fuzzy clustering, we must start with the fully connected network depicted in Fig. 3 or a clustered network with the inter-cluster edges added. We could perform a fuzzy clustering on the entire network, but the result would again be too dense to facilitate exploration. Another approach is to select the nodes from several clusters that are of interest—for example, clusters with high ranking or that show consistently high over-expression or under-expression. In this case, we’ll choose the nodes from nine clusters that contain many over-expressed genes: clusters 13, 19, 20, 25, 82, 197, 242, 259, and 668. We selected the nodes in those clusters by using the *clusterMaker2* “link selection across networks” function and then selecting the clusters in the clustered view (Fig. 6). This selected the corresponding nodes in the full network (Fig. 3). Then we could perform fuzzy clustering by selecting **Apps → clusterMaker Cluster Network → Cluster Fuzzifier**. We chose *stringdb::score* as the **Array Source**, *1/value* as the **Edge weight conversion** (to convert it to a distance) and selected **Cluster only selected nodes** and selected **Create new clustered network** to see the result. To show the relationships between the clusters, we redid the layout using the score as an edge weight and changed the shape of the fuzzy cluster centroids (Fig. 8).

The results indicate that clusters 20, 82, and 197 are closely intertwined. We used the stringApp enrichment analysis to explore the grouping by looking at enrichment of all three clusters, then cluster 20 and 197 together and cluster 82 alone. All three of these clusters represent genes in the mitochondrion exclusively. All of the genes are enriched in the GO Biological Process “Cellular respiration.” Clusters 20 and 197 represent proteins in the TCA cycle, and all of the proteins in cluster 82 are part of the oxidation–reduction process in the mitochondrion, including the mitochondrial respiratory chain complexes III and and IV. It makes perfect sense that genes responsible for cellular respiration would be upregulated in response to heat stress. RIP1, in particular, has been shown to have an important role in the selective export of heat shock RNAs [58]. The close association of these three clusters would not have been apparent without performing a subsequent fuzzy clustering, yet, there is little doubt that cluster 82 is logically separate from clusters 20 and 197, so this is not in any way an indictment of the Leiden cluster results. Furthermore, cluster 20 represents two complexes, the mitochondrial succinyl-CoA synthetase complex with LSC1 and LSC2 and the respiratory chain complex II with SDH1, SDH2, SDH3, SDH4, and YJ045W (SDH9), which is a paralog of SDH1. The separation between cluster 20 and 197 is also explained by the STRING evidence, which shows that while LSC2 has a strong confidence score for interaction with IDH2, it does not bind with any confidence to the rest of the proteins. On the other hand, LSC1 has a strong confidence score for interaction with SDH2, and both LSC1 and LSC2 have moderate confidence scores for interaction with the rest of the proteins in respiratory chain complex II.

Overall, the fuzzy clustering analysis provides us with a much more nuanced view of the relationships between these clusters, allowing a more detailed analysis of the molecular processes of the heat shock response in yeast. The incremental approach—discrete clustering, then fuzzy clustering of the groups of interest—allows us to avoid overinterpreting the initial clustering while not increasing the complexity that would result from fuzzy clustering of the entire network.

### Cluster ranking

The goal of ranking is to order the clusters based on some criteria (typically node attributes) to determine the most relevant or important clusters [39].

**Fig. 9:**
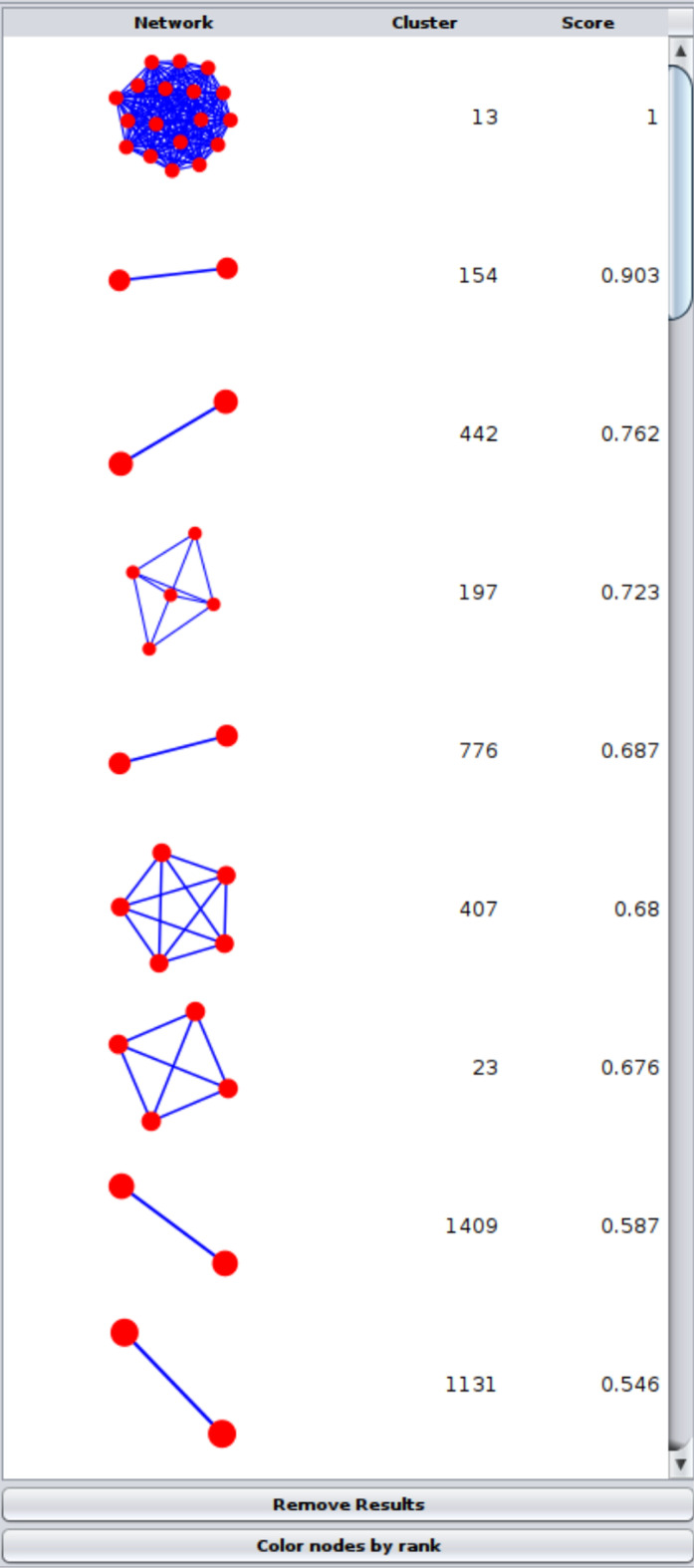
clusterMaker2 ranking panel with ranking results from the multiple nodes and edges (additive sum) ranking algorithm. As discussed in the test, the highest cluster with score 1.0 is cluster 13

After clustering the network with Leiden clustering as shown in Fig. 6, MAA ranking was applied from **Apps → clusterMaker Ranking → Create rank from multiple nodes and edges (additive sum)**. To focus on up-regulated genes, we will choose the same node attributes (GPL51-01–GPL51-08) and select **Basic** normalization, but **Only positive values** for the Two-tailed values normalization. After running the ranking algorithm, the ranking panel **(**Fig. 9**)** can be opened from **Apps → clusterMaker Visualizations → Show results from ranking clusters**. By calculating a ranking score for each cluster, we can analyze the relevance of the clusters in terms of the research question. In this case, higher ranking score would imply that the cluster is more associated with yeast heat shock. The genes grouped in the cluster 13 with a ranking score 1.0 were examined more closely to assess the biological relevance of the ranking results. Cluster 13 was chosen for closer examination because its ranking score was the highest of all clusters.

The biological relevance of cluster 13 was checked in two ways. First, the proteins in the cluster were manually looked up in the protein database UniProt [59]. Furthermore, functional enrichment analysis was performed on the cluster.

Looking at Uniprot, most of the genes in cluster 13 are associated with yeast heat shock, which supports the results of the ranking algorithm. For example, SSA4 (https://www.uniprot.org/uniprot/P22202) and HSP104 (https://www.uniprot.org/uniprot/P31539) are known heat-shock proteins.

**Fig. 10:**
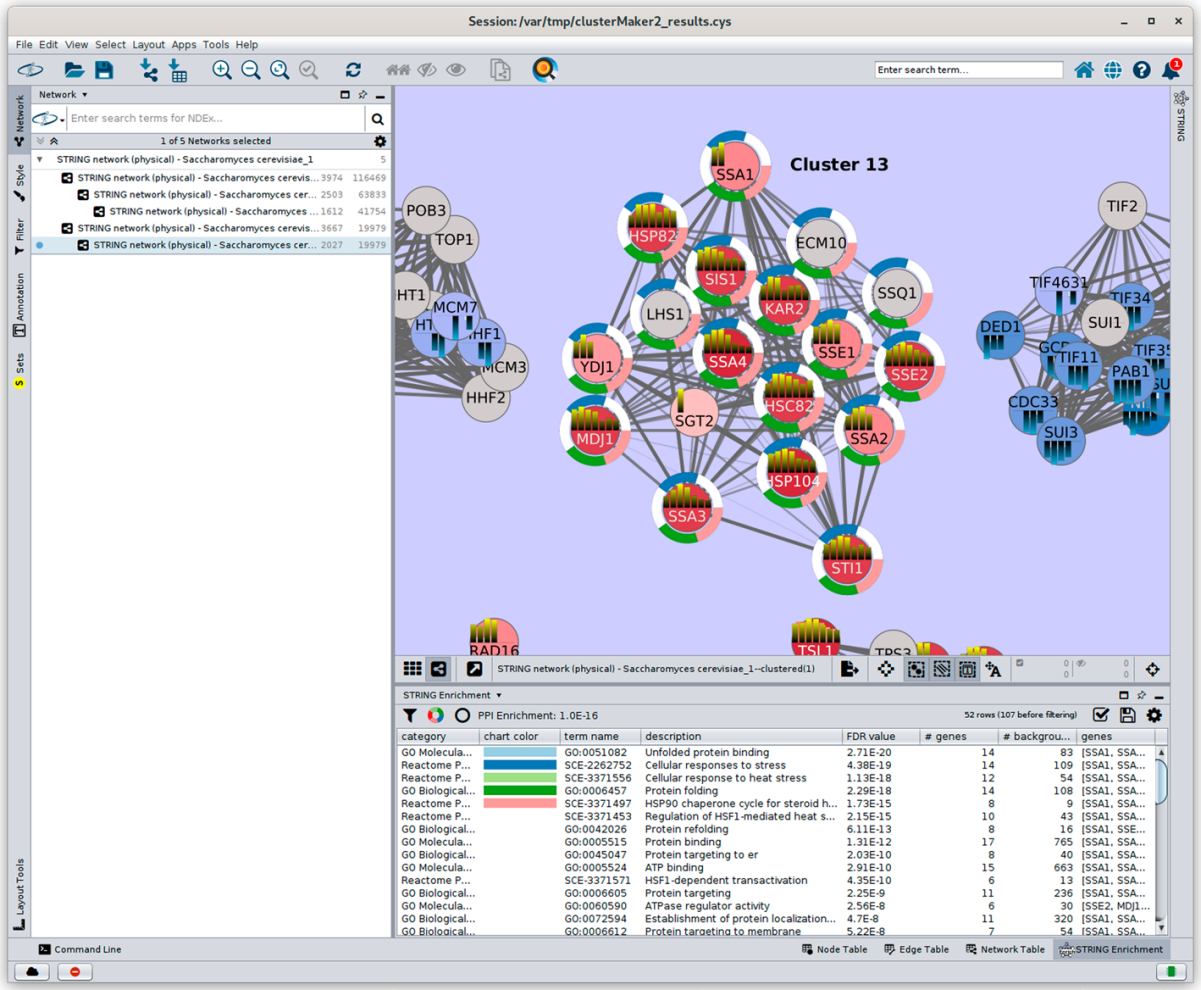
Screenshot of Cytoscape showing functional enrichment results for cluster 13. The top 5 results are shown, which include unfolded protein binding (cyan), cellular response to stress (blue), cellular response to heat stress (light green), protein folding (green), and HSP90 chaperone cycle for steroid hormone receptors (SHR) (pink). The second, third, and fifth terms are Reactome pathways; the first term is from GO molecular function; and the fourth term is from GO biological process. As shown in Fig. 6, the heatstrips represent the individual expression fold change for each time point. The enrichment results strongly correspond to the consistent up-regulation across the entire time spectrum for most of these genes

Functional enrichment analysis was performed on cluster 13 using the STRING Functional Enrichment function [47] (Fig. 10). Most genes in the cluster are associated with GO [60] Molecular function “Unfolded Protein Binding” and GO Biological Process “Protein Folding.” Furthermore, Reactome [61] Pathways “Cellular responses to Stress” and “Cellular Responses to Heat Stress” included most of the proteins involved.

### Assessing the biological relevance of other clusters with high, mid and low scores

Cluster 13 is not the only one with a high ranking score. Cluster 154 has a score of 0.903, which indicates a high relevance as well. The cluster consists of two proteins, which are both strongly upregulated. One of these is a heat shock protein, HSP42, involved in cytoskeleton reorganization after heat shock (https://www.uniprot.org/uniprotkb/Q12329/entry). Cluster 447, with a relatively high score of 0.762, is also a two-protein cluster. Both proteins are upregulated, indicating an association with heat shock. Cluster 259 has a low score of 0.469 and consists of four genes, of which some are up- and some downregulated, and one protein is neutral.

## Discussion

There is little doubt or debate about the importance of grouping data based on some similarity or distance metric. Within a network context, whether the nodes represent genes, proteins, cells, or any other entities linked by some form of relationship, the metric can be expressed as the connectivity or weighted connectivity between the linked entities. Independent of a network context, the metric can be expressed as the similarity of values or features between the entities. However, as demonstrated above, combining grouping based on network connectivity with that based on entity features can be valuable. *clusterMaker2* provides exactly that capability.

However, it is also clear that *clusterMaker2* does not cover the entire space of community detection, clustering, and unsupervised classification. It might be argued that some favored conventional clustering algorithm or dimensionality reduction technique is missing. While this may be true, it is also the case that no package could provide access to all clustering and dimensionality reduction techniques. The new web service framework added in the latest releases of *clusterMaker2* should provide us with the ability to respond much more quickly to user requests and we will continue to add new algorithms over time based on user feedback.

Another potential criticism of *clusterMaker2* is that all of the algorithms implemented in *clusterMaker2* are already available in R, or Python. This is certainly true, and we have taken advantage of that by using Python packages extensively for our web service implementation, so we are extremely grateful and supportive of the R and Python bioinformatics communities. On the other hand, *clusterMaker2* is integrated into Cytoscape and is part of the overall Cytoscape ecosystem. This provides the opportunity to leverage these clustering packages from within Cytoscape without programming, and to integrate those results into Cytoscape networks. As mentioned above, *clusterMaker2* is already used by other Cytoscape apps to add clustering capabilities to specific workflows. Integration into Cytoscape also provides significant advantages in terms of the integration of the visualizations provided with the network, as demonstrated in the figures above. *clusterMaker2* specifically links the visualizations, providing a brushing and linking [62, 63] facility between the various visualizations and the Cytoscape network.

One other criticism is that *clusterMaker2* provides very limited support for cluster evaluation techniques. Currently, only a modularity score is produced for network cluster algorithms and silhouette is provided for algorithms that require a cluster number as an input parameter. However, there are several other cluster evaluation techniques that provide internal or external measures of the clustering. External cluster evaluation techniques such as the Rand Index [64] and Mutual Information [65] require some type of ground truth, which makes them somewhat unsuitable for the typical *clusterMaker2* use cases where ground truth is almost never known. Internal cluster evaluation techniques, on the other hand, do not require a ground truth, and would be worthwhile to additions to a future version of *clusterMaker2.* For example, both the Calinski-Harabasz index [66] and the Davies-Bouldin index [67] could be added to *clusterMaker2* in a new **Cluster Evaluation** category. Until that point, however, the Python package sklearn provides these functions (sklearn.metrics.calinski_harabasz_score and sklearn.metrics. davies_bouldin_score and a good tutorial covering these methods is available on the analyticsindiamag.com web site [68]. It should be noted, however, that while cluster evaluation algorithms provide an assessment of the clustering based on various measures of “goodness”, the best clustering for most *clusterMaker2* users is one that reflects the underlying biology. A good way to inspect that is shown in the workflow above, where over-representation analysis was performed on each cluster to determine the extent to which the underlying biology was reflected in the cluster. This in no way detracts from the use of cluster evaluation algorithms, but they should be used in conjunction with biological meaningful measures. Hopefully, a future version of *clusterMaker2* will facilitate that.

The contributions of supervised and semi-supervised machine learning techniques to biology are also important to mention here. In particular, modern unsupervised learning techniques such as autoencoders have been used effectively in a biological context (c.f. [69–73]), and *clusterMaker2* currently does not provide any support for these algorithms. While it is clear that these techniques are achieving great success, at this point, it would be difficult to incorporate them into *clusterMaker2*’s architecture. However, it should be noted that applications such as AlphaFold [74] have been extremely successful by distributing pre-trained neural networks, skipping the training step for end users. One could imagine similar approaches that would pre-train a series of neural networks for common types of biological networks. Pre-trained networks would be much easier to integrate into *clusterMaker2*.

Finally, there are several areas to explore in future versions of *clusterMaker2*. First, as mentioned above, we continue to respond to user requests for additional clustering algorithms that are currently not included in *clusterMaker2*. Second, we are exploring unsupervised machine learning algorithms such as variational autoencoders [75] for inclusion. Finally, the usability of the scatterplot implementation could be improved to provide support for better selection modes, panning, and zooming.

## Conclusions

*clusterMaker2* represents a significant advance over the previously published version of *clusterMaker* and is the culmination of a large number of improvements, including the addition of dimensionality reduction techniques, fuzzy clustering methods, and the use of web services to improve the ease of implementation of new algorithms and the performance of algorithms on large networks. All of these additional features have been integrated into the Cytoscape ecosystem and have been made accessible via CyREST commands to allow integration into bioinformatic pipelines using R or Python. Most importantly, *clusterMaker2* provides an easy-to-use tool to perform clustering and to visualize clusters within the Cytoscape network context.

### Availability and requirements

Project name: clusterMaker2

Project home page: https://www.rbvi.ucsf.edu/cytoscape/clusterMaker2/

Project source: https://www.github.com/RBVI/clusterMaker2/

Operating system(s): Platform independent

Programming language: Java

Other requirements: Java 11, Cytoscape 3.9 or higher

License: GNU LGPL

Any restrictions to use by non-academics: None.

## Data Availability

The datasets generated and/or analysed during the current study are available publicly as a Cytoscape session file at: https://doi.org/10.5281/zenodo.7366164
